# Intracranial pressure after subarachnoid hemorrhage

**DOI:** 10.1186/cc12279

**Published:** 2013-03-19

**Authors:** A Lombardo, T Zoerle, ER Zanier, L Longhi, N Stocchetti

**Affiliations:** 1 Fondazione IRCCS Ca' Granda-Ospedale Maggiore Policlinico, University of Milan, Italy; 2 Mario Negri Institute, Milan, Italy

## Introduction

Elevated intracranial pressure (ICP) may have deleterious effects on cerebral metabolism and mortality after aneurysmal subarachnoid hemorrhage (SAH) [1,2], but its relevance has not yet been well explored. Aims of this study are to track ICP changes after SAH, to identify clinical factors associated with it and to explore the relationship between ICP and outcome.

## Methods

A total of 116 consecutive SAH patients with ICP monitoring were enrolled. Episodes of ICP >20 mmHg for at least 5 minutes and the mean ICP value for every 12-hour interval were analyzed. The highest mean ICP collected in every patient was identified. ICP values were analyzed in relation to clinical and CT findings; 6-month outcome and ICU mortality were also introduced in multivariable logistic models.

## Results

Eighty-one percent of patients had at least one episode of elevated ICP and 36% had a highest mean ICP >20 mmHg. The number of patients with highest mean ICP >20 mmHg or with episodes of HICP was maximum at day 3 after SAH and decreased only after day 7. Neurological status, aneurysmal rebleeding, amount of blood on CT and CT ischemic lesion occurred within 72 hours from SAH were significantly related to highest mean ICP >20 mmHg in a multivariable model. Patients with highest mean ICP >20 mmHg showed significantly higher mortality in ICU. However, ICP is not an independent predictor of 6 months unfavorable outcome.

## Conclusion

Elevated intracranial pressure is a common complication in the first week after SAH. It is associated with early brain injury severity and ICU mortality.

## References

[B1] Heuer GG Relationship between intracranial pressure and other clinical variables in patients with aneurismal subarachnoid hemorrhage J Neurosurg 2004 101 408 416 10.3171/jns.2004.101.3.0408 15352597

[B2] Nagel A Relevance of intracranial hypertension for cerebral metabolism in aneurismal subarachnoid hemorrhage. Clinical article J Neurosurg 2009 111 94 101 10.3171/2009.1.JNS08587 19284237

